# Latitudinal variation of leaf stomatal traits from species to community level in forests: linkage with ecosystem productivity

**DOI:** 10.1038/srep14454

**Published:** 2015-09-25

**Authors:** Ruili Wang, Guirui Yu, Nianpeng He, Qiufeng Wang, Ning Zhao, Zhiwei Xu, Jianping Ge

**Affiliations:** 1 Synthesis Research Center of Chinese Ecosystem Research Network, Key Laboratory of Ecosystem Network Observation and Modeling, Institute of Geographic Sciences and Natural Resources Research, Chinese Academy of Sciences, Beijing 100101, China; 2 University of Chinese Academy of Sciences, Beijing 100049, China; 3 College of Life Sciences, Beijing Normal University, Beijing 100875, China

## Abstract

To explore the latitudinal variation of stomatal traits from species to community level and their linkage with net primary productivity (NPP), we investigated leaf stomatal density (SD_L_) and stomatal length (SL_L_) across 760 species from nine forest ecosystems in eastern China, and calculated the community-level SD (SD_C_) and SL (SL_C_) through species-specific leaf area index (LAI). Our results showed that latitudinal variation in species-level SD_L_ and SL_L_ was minimal, but community-level SD_C_ and SL_C_ decreased clearly with increasing latitude. The relationship between SD and SL was negative across species and different plant functional types (PFTs), but positive at the community level. Furthermore, community-level SD_C_ correlated positively with forest NPP, and explained 51% of the variation in NPP. These findings indicate that the trade-off by regulating SD_L_ and SL_L_ may be an important strategy for plant individuals to adapt to environmental changes, and temperature acts as the main factor influencing community-level stomatal traits through alteration of species composition. Importantly, our findings provide new insight into the relationship between plant traits and ecosystem function.

Changes in the environment can affect ecosystem processes and function directly through influences on abiotic controls, and indirectly through influences on the physiology, morphology, behavior of individual plants, the structure of populations, and the composition of communities^1^,^2^,^3^. Recent studies have suggested that the spatial and temporal variation of ecosystem functions is difficult to be explained sufficiently by classical climatological or biogeographical approaches, and plant traits have the potential to advance the understanding in this field^2^,^4^. Plant traits have been well recognized as important determinants of ecosystem function^2^,^5^, and it is possible to aggregate functional traits measured on organisms to explain the function of populations, communities, ecosystems, and beyond^2^,^4^,^5^,^6^. Thus, understanding plant traits and their controlling factors at a large scale is of importance to explore how organisms and environmental conditions collectively shape the variation of ecosystem function in space and time^2^, and to predict ecosystem function in response to environmental changes.

Spatial and temporal variation of ecosystem function, such as net primary productivity (NPP), evapotranspiration, and water use efficiency, has received much attention to date^2^,^7^,^8^. Some studies have demonstrated that leaf traits, including leaf size, specific leaf area (SLA), and tissue nitrogen and phosphorus concentration, are strongly correlated with ecosystem productivity^2^,^3^,^5^. Abiotic factors (e.g., soil, climate, and disturbance) may exert indirect effects on the variation of NPP through regulating leaf area and duration of photosynthetic season^3^. Leaf stomata regulate gas exchange between plants and the atmosphere, and thus control the balance between water loss and CO_2_ uptake to some extent^9^. Compared with leaf morphological and chemical traits, stomatal traits are more useful indicators to investigate the process of photosynthesis and evapotranspiration. Among stomatal traits, stomatal density (SD) and stomatal size (or length, SL) are considered as key morphological traits to jointly determine stomatal conductance of leaves to CO_2_ and H_2_O^9^,^10^,^11^.

Biogeography and variability of stomatal traits of leaves at a large scale are highly important but currently poorly understood. Most of previous studies used short-term control experiments to investigate the responses of stomatal morphological traits to changing environments, such as CO_2_ concentration^12^, light intensity^13^, temperature^14^, and water status^15^,^16^. The response of SD to elevated temperature is inconsistent, as it increases^17^, decreases^14^, or remains unchanged^18^. Furthermore, SD is also affected by water availability, and moderate water deficits have a positive effect on SD^15^,^16^. These observations focus on the responses or plasticity of stomatal traits to controlled stresses at the local scale, but they can’t clarify the variation of stomatal traits at a large scale as a result of long-term adaption to changing environments^19^. Recently, Yang *et al.*^20^ reported that lower temperature and higher insolation led to higher SL but lower SD of grassland species in the Tibetan Plateau compared to Inner Mongolia. Stenstrom *et al.*^21^ found that the SD of *Carex* increased and SL decreased with increasing temperature and precipitation in arctic Eurasia. However, how to scale up the stomatal traits from species to community level and how to establish the relationship between these traits and ecosystem function are still important challenges.

At the leaf level, short-term field measurements have suggested that a positive relationship exists between the maximum stomatal conductance (determined by the size and density of stomata) and the photosynthetic capacity^9^. Given that ecosystem function is manifested through the plant community rather than individual plants or species, it is expected that ecosystem NPP is closely related to community-level stomatal traits. In this study, through an investigation of SD and SL across 760 common species (to differentiate from community-level stomatal traits, we used SD_L_ and SL_L_ to represent leaf or species-level stomatal traits thereafter) in nine typical forests, ranging from tropical monsoon rainforest to cold-temperate coniferous forest along the north-south transect of eastern China (NSTEC, Fig. 1 and Supplementary Table S1), we explored the latitudinal patterns of leaf stomatal traits and their main controlling factors at the species and community levels. Furthermore, we tested the assumption that stomatal traits at the community level (SD_C_ and SL_C_) would be closely related to NPP in forests at a large scale.

## Results

### Overall statistics of stomatal traits

Across all 760 species, the mean values of species-level SD_L_ and SL_L_ were 219.15 stomata mm^–2^ and 28.16 μm, with the range from 24.82 to 1,371.93 stomata mm^–2^ for SD_L_ and 9.34–93.76 μm for SL_L_, respectively (Fig. 2). Moreover, the variation of SD_L_ was larger than that of SL_L_ (coefficient of variation, CV = 0.70 vs. 0.38, Fig. 2). For nine forest communities, the mean values of community-level SD_C_ and SL_C_ were 1,497.09 stomata mm^−2^ and 36,032.13 μm mm^–2^ (see Supplementary Table S2), with CVs of 0.55 and 0.49, respectively.

SD_L_ and SL_L_ varied markedly across different plant functional types (PFTs) (Fig. 3 and Supplementary Fig. S1). Compared with shrubs and herbs, the leaves of trees had denser but smaller stomata (*F *= 234.80, *P *< 0.001 for SD_L_; *F *= 185, *P *< 0.001 for SL_L_). Among trees, broadleaves had higher SD_L_ and lower SL_L_ than conifers (*F *= 13.48, *P *< 0.001 for SD_L_; *F *= 11.73, *P *= 0.001 for SL_L_), and evergreen broadleaves had higher SD_L_ and less SL_L_ than those of deciduous counterparts (*F *= 24.10, *P *< 0.001 for SD_L_; *F *= 57.83, *P *< 0.001 for SL_L_).

### Relationships between SD and SL at the species and community levels

Strong negative relationship between SD_L_ and SL_L_ was observed across species (Fig. 4A) and different PFTs (Fig. 5), in which SL_L_ decreased linearly with the increase in SD_L_ (after log_10_ transformation). The results of standardized major axis (SMA) tests showed that the slopes differed significantly among growth forms (*P *< 0.05, Fig. 5A and Supplementary Table S3), and the slope for herbs was steeper than those of trees or shrubs. SMA tests for common slopes revealed no significant difference between evergreen and deciduous trees (*P *> 0.05), but difference was observed in the y-axis intercept, with higher SL_L_ at any given SD_L_ in deciduous trees than that in evergreen trees (*P *< 0.05, Fig. 5B and Supplementary Table S3).

In contrast to the results at the species and PFT levels, a significantly positive relationship between SD_C_ and SL_C_ was observed at the community level (R^2 ^= 0.97, *P *< 0.001, Fig. 4B).

### Latitudinal variation in stomatal traits and their influencing factors

At the species level, plant species at lower latitudes had higher SD_L_ than those distributed in higher latitudes (R^2 ^= 0.08, *P *< 0.001, Fig. 6A), whereas the observed latitudinal trend of SL_L_ was opposite (R^2 ^= 0.07, *P *< 0.001, Fig. 6A). When scaling up to the community level, SD_C_ and SL_C_ decreased nonlinearly with increasing latitude (R^2 ^= 0.83, *P *< 0.05 for SD_C_; R^2 ^= 0.76, *P *< 0.05 for SL_C_, Fig. 6B).

Mixed-effect models were used to explain the effects of environmental factors and PFT on the stomatal traits (Table 1 and Supplementary Table S5). At the species level, the best model for SD_L_ included PFT, mean annual temperature (MAT), soil N, and their interactions as predictors; the best model for SL_L_ only included PFT and MAT (Supplementary Table S5). PFT could explain the largest part of the variation in SD_L_ and SL_L_ (15.3–22.8%) at the species level, and the environmental variables accounted for only a small proportion (4.7–5.4%, Table 1), although the relationships between MAT or soil N and stomatal traits were significant (Fig. 7A–C). In addition, the interactions among PFT, MAT and soil N accounted for a large proportion of the variation in SD_L_ (16.6–17.9%, Table 1).

To partition the variance of SD_L_ and SL_L_ into among-site and within-site components, the data were further analyzed through nested random-effect analysis of variance (ANOVA), with species nested within sites. We found that >50% of the variance in SD_L_ and SL_L_ existed within sites, and 10.3–13% of variance occurred across sites (Supplementary Table S6).

At the community level, both SD_C_ and SL_C_ increased significantly with the increase in MAT (*P *< 0.05, Fig. 7D,E). The best models of SD_C_ and SL_C_ only included MAT (Supplementary Table S5), which accounted for 35.2–36.7% of total variation (Table 1). Furthermore, the site explained 30.9% and 45.9% of the variation in SD_C_ and SL_C_, respectively (Table 1).

### Relationships between community-level stomatal traits and NPP

Community-level SD_C_ and SL_C_ were closely correlated with forest NPP (Fig. 8). NPP increased linearly with increasing SD_C_ and SL_C_ (*P *< 0.05, Fig. 8). Moreover, in the general linear model analysis, SD_C_ explained 51% of the total variation in forest NPP at the large scale (Supplementary Table S7), whereas SL_C_ did not have a significant effect on NPP (*P *> 0.05, Supplementary Table S7), probably because of the high self-correlation between SD_C_ and SL_C_ (R^2 ^= 0.97, Fig. 4B).

## Discussion

This study is the first to explore the latitudinal variation in stomatal traits at the species and community levels from tropical monsoon rainforest to cold-temperate coniferous forest. Furthermore, our findings establish a relationship between stomatal traits and the spatial variation in ecosystem NPP, and thus provide useful biological information for modeling ecosystem carbon and water cycles under global changes.

As expected, community-level stomatal traits were closely related to forest NPP in our study (Fig. 8). In previous studies, tight relationships between aboveground NPP and other community-aggregated traits (such as SLA and leaf N) have been documented^5^,^22^. Community structure is the basis for scaling up plant traits from species to community level^5^. Furthermore, leaf area index (LAI) has been widely considered as an important parameter controlling the interception of light and water, transpiration, photosynthesis, carbon, and nutrient cycles^3^. In this study, we developed a new methodology to determine community-level stomatal traits through leaf-level measurements weighted by species-specific LAI. Such defined community-level stomatal traits were useful to qualify the total stomatal number and length per unit ground surface area within communities, and could reflect the potential to exchange water vapor and CO_2_ between plant community and the atmosphere. The results from Fig. 8 and Supplementary Table S7 showed that community-level SD_C_ positively correlated with forest NPP and accounted for 51% of spatial variation in NPP along the NSTEC. These findings suggest that the stomatal data measured at the species level may be associated with NPP through community structure, which provides new evidence for the linkage between plant traits and ecosystem function^2^. Moreover, community-level SD_C_ can better characterize the capacity of CO_2_ and H_2_O exchange between plant community and atmosphere, and thus may be a new ecological parameter in modeling CO_2_ and H_2_O exchange at the community scale.

Because of measurement difficulties and other limitations, few studies have systematically analyzed the biogeographical variation in stomatal traits across wide gradients of environmental factors. In this study, we found that latitudinal variation in species-level SD_L_ and SL_L_ was minimal (R^2^ ranging from 0.07 to 0.08, Fig. 6A), and environmental variables weakly modulated stomatal traits (4.7–5.4%, Table 1). The main reason for these weak correlations was that a substantial proportion of the variation in SD_L_ and SL_L_ was observed within sites (>50%, Supplementary Table S6) and thus resulted in relatively weak spatial patterns of species-level stomatal traits along the large-scale environmental gradients. Similarly, high within-site variation is normal for global-scale analyses of plant morphological, chemical, anatomical, and physiological traits^23^,^24^. The large variance explained by within-site differences implies that site scale (or community level) is of particular relevance when studying mechanisms of species assembly^25^.

Changes in PFTs were dominant factors influencing the biogeographic distribution of species-level stomatal traits, explaining the largest part of the variation in species-level SD_L_ and SL_L_ (Table 1). Considerable variation was observed among different PFTs (Fig. 3), reflecting the differences in adaptive strategies of PFTs to their external environment. For example, smaller and denser stomata in trees are associated with higher stomatal conductance and transpiration rates^10^,^26^, which benefits water and nutrient transmission through longer xylem pathways in woody plants^27^. In contrast, large stomata are critical for herbs to optimize light capture in the light-limited environment^9^. Differences in stomatal traits between broadleaves and conifers reflect the differences in leaf vascular architecture, where broadleaved angiosperms reach high stomatal conductance with numerous small stomata, and conifers obtain lower stomatal conductance with fewer large stomata^28^. Compared with deciduous trees, evergreen broadleaves with high leaf SD_L_ can likely acquire high diffusive conductance for water vapor or CO_2_, thereby meeting the high transpiration requirement and simultaneously providing efficient cooling of the leaf^9^,^29^. Thus, changes in PFTs along environmental gradients contributed substantially to generating the observed variation in SD_L_ and SL_L_.

Shifts of PFTs accounted for the major part of the latitudinal variation in SD_L_ and SL_L_; however, climate and soil, especially MAT and soil N (Fig. 7A–C), may directly or indirectly influence stomatal traits by shaping the vegetation biogeography (changes in species composition)^3^,^30^. In this study, the interactive effect of MAT and PFT accounted for 16.6% of the variation in SD_L_, and that of PFT and soil N explained another 17.9% of the variation (Table 1).

In this study, the latitudinal trends of SD_C_ and SL_C_ were significant at the community level (*P *< 0.05, Fig. 6B). Both SD_C_ and SL_C_ decreased with latitude (Fig. 6B), and could be explained largely by MAT (Table 1 and Supplementary Table S5). These results indicated that through regulation of species composition and PFTs within a community, temperature acted as the main environmental factor driving the latitudinal patterns of community-level stomatal traits, even though the influence was moderate at the species level. On the one hand, evergreen broad-leaved forests with higher LAI and SD_L_ also have higher community-level SD_C_ and SL_C_, allowing them to attain effectively high stomatal conductance to meet the high requirements of transpiration and photosynthesis at low latitudes^3^. On the other hand, coniferous forests dominate at high latitude and cold habitats with low SD_L_ (thus low SD_C_ and SL_C_) that allows them to minimize water loss by transpiration and mitigate water-deficit stress resulting from low air and soil temperature^31^.

Stomatal morphological traits and behaviors collectively determine the balance of CO_2_ uptake for plant photosynthesis against water loss by transpiration^9^. In addition to the short-term dynamics resulting from altering the width of the stomatal pore apertures, plants can respond to environmental change by adjusting their stomatal morphology via long-term evolution and development. In this study, SL_L_ correlated negatively with SD_L_ across all species and PFTs (Figs 4A and 5), which was consistent with a general relationship reported by previous studies across multiple species and geological time scales^9^,^10^,^11^. The trade-off between SD_L_ and SL_L_ can be partially explained by physical and energetic constraints^11^. The physical (space) constraints hypothesis assumes that plants adjust their stomatal size and density to optimize stomatal conductance while satisfying a given stomata-to-pavement cell ratio, and thus the allocation of leaf epidermal space to stomata is limited. The second plausible hypothesis for the trade-off between SD_L_ and SL_L_ is the energetic constraint, which emphasizes maximizing the return in terms of stomatal conductance and photosynthesis for a given investment in the construction of stomatal complexes^11^. High stomatal conductance may be related to increased metabolic cost, which may explain why some species revert to producing fewer but larger stomata, particularly for plants grown in low light or low nitrogen availability with a low requirement for stomatal conductance and photosynthesis^11^.

The negative relationship between SD_L_ and SL_L_ governs short-term (plastic) and long-term (evolutionary) adaptations of plant physiological activities to the environment^11^. In contrast, a positive relationship between SD_C_ and SL_C_ was observed at the community level, indicating that the general trade-off between species-level SD_L_ and SL_L_ did not exist between the community-level SD_C_ and SL_C_. Therefore, we assume that the trade-off mechanism through regulation of SD_L_ and SL_L_ may be an important strategy at the species level for adaptation to the external environment, while the adaptation of community-level stomatal traits is achieved mainly through the modulation of community composition. This mechanism could compensate for the limitation of biological conservatism resulting from the trade-off between leaf SD_L_ and SL_L_ at the species level.

In conclusion, community-level SD_C_ and SL_C_ decreased significantly as latitude increased, although latitudinal variation in species-level SD_L_ and SL_L_ was minimal. The adaptive strategies of stomatal traits to the external environment may be different between the species and community levels. We assume that the trade-off between SD_L_ and SL_L_ represents adaptive mechanism of leaf stomata to respond to environmental changes at the species level. While community-level stomatal traits regulate species composition to maximize ecosystem productivity in a given environment, and to some extent compensate for the limitation of biological conservatism resulting from the trade-off between SD_L_ and SL_L_. More importantly, SD_C_ could explain 51% of the variation in forest NPP, which bridges stomatal traits to ecosystem function. Finally, the new framework, which scales up stomatal traits from species to community level weighted by LAI, makes it possible to better parameterize the complex models pertaining to ecosystem carbon and water cycles in the future.

## Methods

### Site description

**Figure 1:**
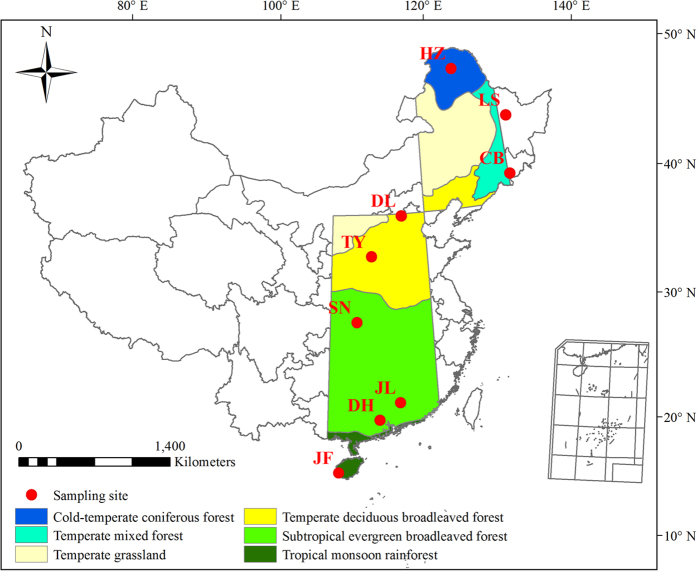
Geographic locations and vegetation types of the sampling sites. JF, Jianfengling; DH, Dinghu; JL, Jiulian; SN, Shennongjia; TY, Taiyue; DL, Dongling; CB, Changbai; LS, Liangshui; HZ, Huzhong. The north-south transect of eastern China (NSTEC) is highlighted with different colors representing different vegetation types^32^. The map is generated using ArcGIS 10.0 software.

**Figure 2:**
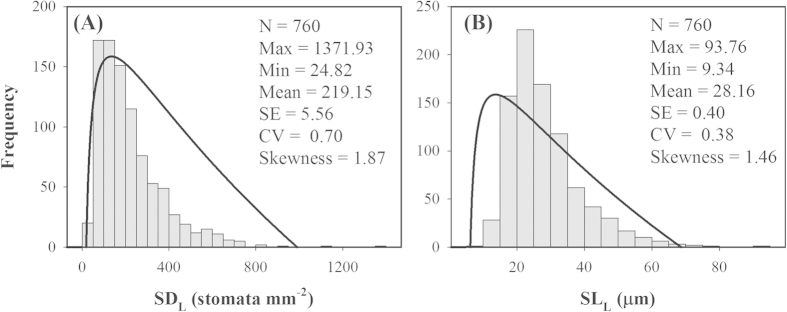
Statistics of stomatal density (SD_L_, (A)) and stomatal length (SL_L_, (B)) across 760 species. N, species number; Max, maximum; Min, minimum; SE, standard error; CV, coefficient of variation.

**Figure 3:**
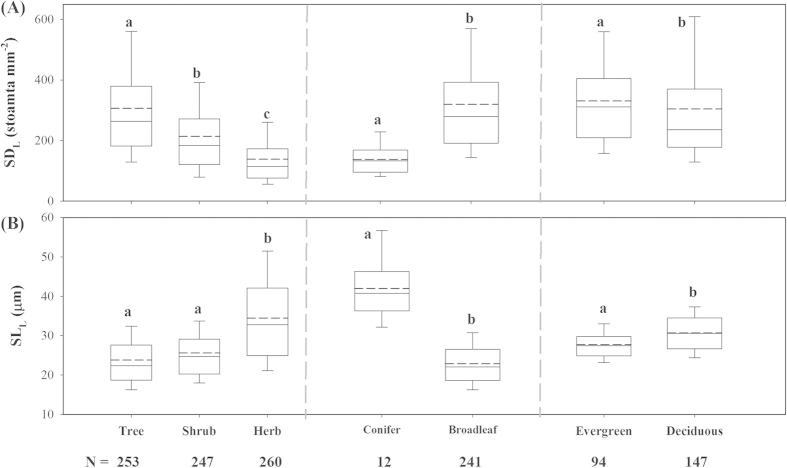
Box plots of stomatal density (SD_L_, (A)) and stomatal length (SL_L_, (B)) across different plant functional types (PFTs). The solid and dashed lines across the middle of the box are mean and median values, respectively. N, species number. Statistical differences (*P *< 0.05) are denoted by different letters using one-way analysis of variance (ANOVA).

**Figure 4:**
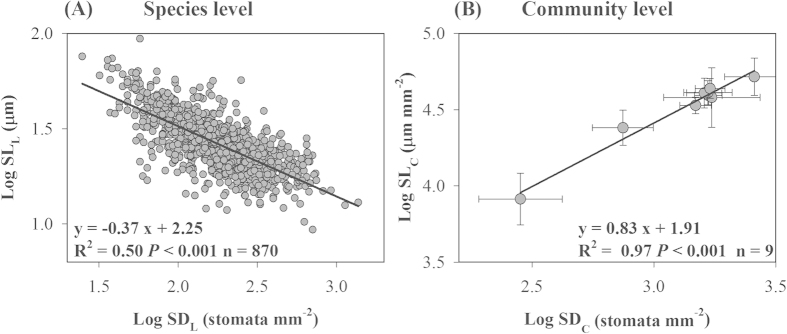
Relationships between stomatal density (SD) and stomatal length (SL) at the species (**A**) and community levels (**B**). n, number of observations. Error bars in panel (**B**) represent ± 1 standard error.

**Figure 5:**
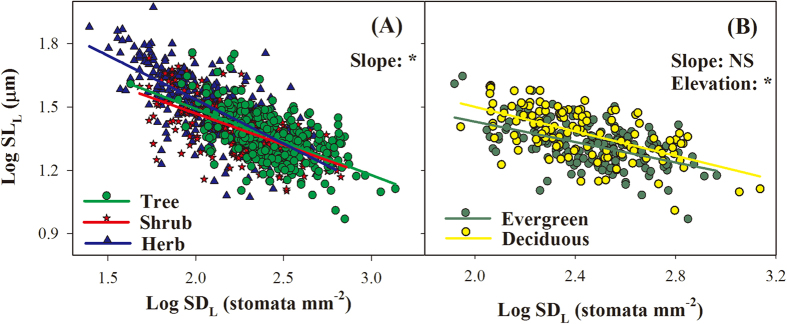
Standardized major axis (SMA) regressions between species-level stomatal density (SD_L_) and stomatal length (SL_L_) across different growth types (**A**) and leaf habits (**B**). “Slope”, difference in SMA slopes; “Elevation”, difference in SMA elevations (i.e. y-axis intercept); *significantly different (*P *< 0.05); NS, not significantly different (*P *> 0.05). Sample size and results of regression analyses are presented in Supplementary Table S3.

**Figure 6:**
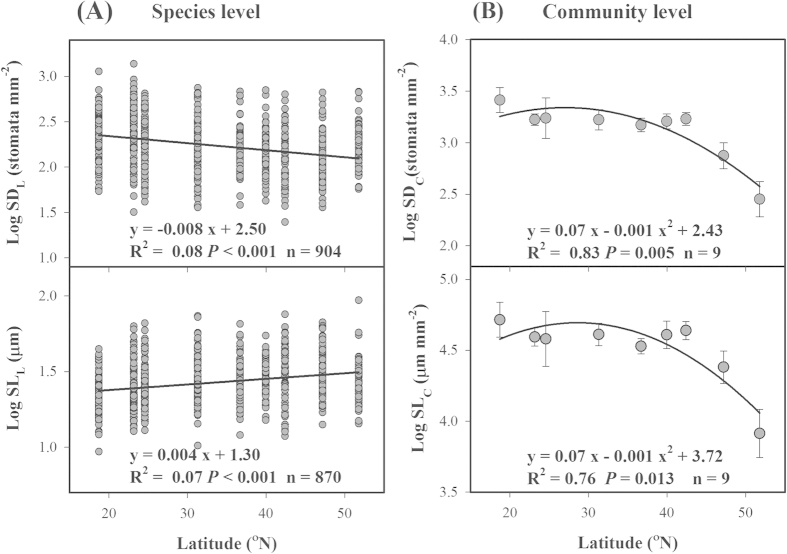
Latitudinal trends of stomatal density (SD) and stomatal length (SL) at the species (**A**) and community levels (**B**). n, number of observations. Error bars in panel (**B**) represent ± 1 standard error.

**Figure 7:**
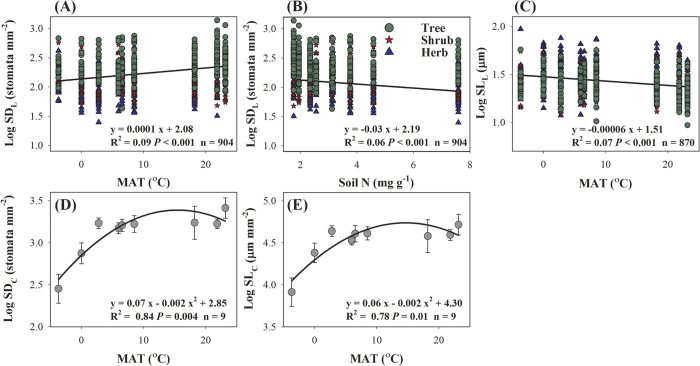
Relationships between stomatal traits and environmental variables. SD_L_, SL_L_, SD_C_, and SL_C_ are the stomatal density and length at the species and community levels, respectively. Environmental variables include mean annual temperature (MAT), mean annual precipitation (MAP), and soil N. n, number of observations. Error bars in panels (**D**) and (**E**) represent ± 1 standard error.

**Figure 8:**
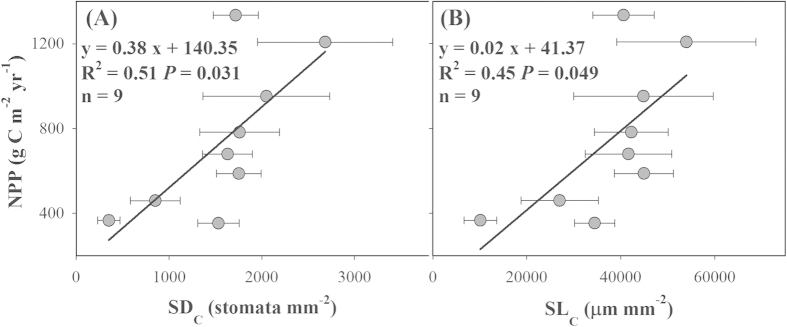
Relationships between net primary productivity (NPP) and community-level stomatal density (SD_C_, (A)) and stomatal length (SL_C_, (B)). n, number of observations. Error bars represent ± 1 standard error.

The NSTEC is a unique forest belt driven mainly by a thermal gradient, and includes almost all forest types found in the northern hemisphere^32^ (Fig. 1); therefore, it offers an ideal condition to test the ecological and evolutionary responses of plants to environmental changes^32^. Nine natural forests along the NSTEC were selected to conduct field sampling, including Jianfengling (JF), Dinghu (DH), Jiulian (JL), Shennongjia (SN), Taiyue (TY), Dongling (DL), Changbai (CB), Liangshui (LS), and Huzhong (HZ) (Fig. 1 and Supplementary Table S1). These ecosystems span latitudes from 18.7 to 51.8 °N, with MAT ranging from −3.67 to 23.15 °C and mean annual precipitation (MAP) ranging from 472.96 to 2265.80 mm. The soil type varies from tropical red soils with low organic matter to brown soils with high organic matter. Correspondingly, vegetation type varies among tropical monsoon rainforest, subtropical evergreen forest, temperate deciduous forest, and cold-temperate coniferous forest. To minimize anthropogenic disturbances, we set up the sampling plots within well-protected national nature reserves in each forest type, where the vegetation is relatively homogenous and is strongly representative of the given forest ecosystems.

### Field sampling and measurement

The field survey was conducted during July and August of 2013. Three or four experimental plots (30 × 40 m) were set up in each forest ecosystem. The geographic information (latitude, longitude, and altitude), plant species identity, and community structure were determined for each plot. The number, height, diameter at breast height (DBH) of all trees with DBH ≥ 2 cm (basal stem diameter for shrubs), and aboveground live-biomass of all herbs were measured. Subsequently, 20 fully expanded sun leaves were collected from four individuals of each plant species using a long chain saw or by two professional climbers, and all of the leaf samples for a given plant species from one plot were mixed together as a replicate. Collected fresh leaves were stored in sealed plastic bags and scanned to determine leaf area within 4–8 h after collection. In total, 904 species-at-site combinations, consisting of 760 plant species from 427 genera and 154 families, were collected. Species were also grouped in different PFTs by the possible groups: growth form (herbs, shrubs, and trees), leaf type (coniferous vs. broadleaved trees), and leaf habit (evergreen vs. deciduous broadleaved trees). At each plot, soil samples (0–10 cm) were collected randomly from 30–50 points using a 6-cm-diameter auger, and then mixed thoroughly.

We randomly selected ten fresh leaves for each plant species and scanned them using a scanner (CanoScan LiDE 110, Japan), then measured the area by using Photoshop CS (Adobe Systems, San Jose, USA). Finally, the leaves were oven-dried at 70 °C for 48 h and then weighed to calculate SLA (m^2^ kg^−1^)^33^.

Leaf SD_L_ (stomata mm^–2^) and SL_L_ (μm) were measured from surface impressions of the mid-blade abaxial leaf surface made with clear nail polish (methods described in Wang *et al.*^34^). All stomatal measurements were conducted with electronic image analysis equipment (COIC XSZ-HS3 and MIPS software, Optical Instrument Co., Ltd., Chongqing, China). In total, 4284 leaf cuticle images were obtained (see examples in Supplementary Fig. S1).

### Calculation of community-level stomatal traits

Here, we defined the total stomata number per unit ground surface area within a community as community-level SD_C_ (stomata mm^–2^) and total stomatal length per unit land area as community-level SL_C_ (μm mm^–2^). Community-level stomatal values were calculated as the sum of species-specific SD_Li_ and SL_Li_ weighted by their LAI (m^2^ m^–2^) in each plot (30 × 40 m). To estimate LAI, we firstly calculated the foliar biomass of woody species based on the mean DBH (basal stem diameter for shrubs) and height through allometric equations, which were obtained from Chinese Ecosystem Research Net (CERN) database (http://159.226.111.42/pingtai/cernc/index.jsp), published studies, and our previous field measurements (unpublished data) (available in Supplementary Table S8). The use of species-specific equations is preferred in the calculation of foliar biomass; however, this is virtually unrealistic in practice, especially for tropical rainforests. Therefore, we used the biomass equations from the same genera, similar PFT, or mixed-species equations of a forest to estimate species’ foliar biomass when their allometric equations were unavailable. In total, 246 species-specific equations with R^2^-values ranging from 0.52 to 1.00 were used in this study. For herbs, the foliar biomass was represented by aboveground biomass. Then, species-specific foliar biomass multiplied by SLA was used to calculate the LAI_i_^35^. Finally, community-level SD_C_ and SL_C_ were calculated according to equations (1) and (2). The frameworks to scale up stomatal traits from species to community level were also illustrated in Supplementary Fig. S2.


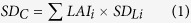



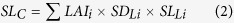


### Ecosystem NPP

The ecosystem NPP (g C m^–2^ yr^–1^) used in this study was derived from Luo^36^, who estimated the biomass and NPP of 668 stands from 7 major forest types in China. In Luo’s published dataset, NPP was computed as the sum of the annual net increment of root, stem, branch and leaf components of trees, and those of shrubs and herbs. For trees, the annual net increments of stems, branches and roots were obtained by multiplication of the biomass of different components and their growth rate. The annual net increment of leaves was estimated as the leaf biomass divided by leaf age of different trees. The average NPP of the shrub and herb layers was estimated by dividing their biomass by average stand age. A more detailed description to estimate forest NPP is presented in Ni *et al.*^35^ and Luo^36^. From this dataset, we extracted NPP data based on latitude, longitude, and forest type. Because NPP data in Luo’s dataset was based on dry matter, the dry matter was converted into units of C, using a conversion factor of 0.5^37^.

To test the accuracy of these data, we compared ecosystem NPP and LAI with the MODIS products in a 1 × 1 km grid (http://daac.ornl.gov/cgi-bin/MODIS/GLBVIZ_1_Glb/modis_subset_order_global_col5.pl). MODIS NPP data from 2000 to 2010 and LAI data from July to August of 2013 for nine sampling sites were selected. The observed closely relationships between measured NPP and LAI and MODIS products proved our data reliable to some extent (Supplementary Fig. S3).

### Climate data

The climatic variables in this study, including MAT and MAP, were extracted from the meteorological database produced by CERN^38^. The climate dataset at a 1 × 1 km spatial resolution was generated from the data of 740 climate stations of the China Meteorological Administration during 1961 and 2007, using the interpolation software of ANUSPLIN.

The monthly averaged insolation (kWh m^–2^ day^–1^) of nine sampling sites over a 22-year period (1983–2004) was obtained from the NASA Surface Meteorology and Solar Energy dataset (http://eosweb.larc.nasa.gov/sse/) to represent light intensity in this study.

### Analysis of soil properties

Soil water content (SWC, %) was determined after fresh soil was dried at 105 °C for 24 h. Other fresh soil samples were air-dried and sieved with the roots removed by hand, then ground to pass through a 2-mm sieve. Soil total N concentration (mg g^–1^) was analyzed using an elemental analyzer (Vario MAX CN Elemental Analyzer, Germany). Soil total P concentration (mg kg^–1^) was measured by the ammonium molybdate method using a continuous-flow analyzer (AutoAnalyzer3 Continuous-Flow Analyzer; Bran Luebbe, Germany) after H_2_SO_4_-H_2_O_2_-HF digestion^39^.

### Data analysis

Stomatal data were log_10_-transformed prior to analyses to obtain the approximate normality and homogeneity of residuals. We analyzed stomatal traits at the species level (averaged within species at each site) and community level (weighted by species-specific LAI), respectively.

One-way ANOVA with Tukey’s post-hoc test was used to compare differences in species-level SD_L_ and SL_L_ among different PFTs. Then, the bivariate analyses of SD_L_–SL_L_ and SD_C_–SL_C_ relationships were performed using ordinary least squares (OLS) linear regressions, and differences in the slopes and elevations of SD_L_–SL_L_ relationships among PFTs were evaluated by SMA estimation with the R package smatr. In SMA analyses, we tested the heterogeneity of regression slopes, and estimated the common slopes when non-heterogeneity of the slopes was demonstrated (*P *> 0.05). Then, differences in the elevation of regression slopes (i.e. y-axis intercept) were tested using ANOVA with Tukey’s post-hoc tests.

To explore the latitudinal patterns of stomatal traits and their environmental drivers, we performed regression analyses between stomatal traits and latitude at the species and community levels, respectively. Then, we analyzed each of the stomatal traits using linear mixed-effect models with the residual maximum likelihood (REML) method in the R package lme4. In these analyses, we treated environmental variables and PFT as fixed effects, and the random-effect term for site was included to account for the non-independence of species occurring at the same site^23^. This term also allowed us to determine how variance in stomatal traits was partitioned within and across sites. To avoid problems of collinearity among the environmental variables, only variables with insignificant Pearson’s correlation (*P *> 0.05, Supplementary Table S4) were included. The environmental variables that had significant effects (*P *< 0.05) on stomatal traits and interaction terms between PFT and the environmental variables were included in the final model (Supplementary Table S5). Models with lower Akaike’s Information Criterion (AIC) values were chosen as the final best-fit models^40^.

Finally, linear regressions were performed to describe the relationships between ecosystem NPP and community-level SD_C_ and SL_C_, and the relative contributions to the spatial variation of NPP were assessed by a general linear model.

All analyses were conducted using SPSS 13.0 statistical software (SPSS Inc., Chicago, IL, USA, 2004) and R software (version 2.15.2, R Development Core Team 2012).

## Additional Information

**How to cite this article**: Wang, R. *et al.* Latitudinal variation of leaf stomatal traits from species to community level in forests: linkage with ecosystem productivity. *Sci. Rep.* **5**, 14454; doi: 10.1038/srep14454 (2015).

## Supplementary Material

Supplementary Information

Supplementary Dataset 1

## Figures and Tables

**Table 1:** Best mixed models for ecological effects on stomatal density (SD) and stomatal length (SL) at the species and community levels.

Factor	Species level						Community level					
	Log SD_L_			Log SL_L_			Log SL_C_			Log SD_C_		
	*df*	*F*	Variance explained (%)	*df*	*F*	Variance explained (%)	*df*	*F*	Variance explained (%)	*df*	*F*	Variance explained (%)
PFT	4	2.85	15.3*	4	56.70	22.8**						
Environmental variables												
MAT	1	0.51	5.2	1	10.15	5.4*	1	8.16	36.7*	1	6.12	35.2*
Soil N	1	0.12	4.7									
Interaction terms												
PFT × MAT	4	2.89	16.6*									
PFT × Soil N	4	2.62	17.9*									
MAP × Soil N	1	2.14	<0.001									
Random factor												
Site	8	0.46	0.3	8	0.19	0.2	8	7.99	30.9*	8	9.57	45.9**
Residuals	880		40.0	856		71.6	22		32.4	22		18.9

PFT, plant functional type; MAT, mean annual temperature; MAP, mean annual precipitation. *df*, degrees of freedom. Model with the lower Akaike information criterion (AIC) value was chosen as the final model (see Supplementary Table S5). *P < 0.05; **P < 0.01.
