# Supplementary material for: Latitudinal variation of leaf stomatal traits from species to community level in forests: linkage with ecosystem productivity
Source: Sci Rep. 2015 Sep 25;5:14454. doi: 10.1038/srep14454 (PMC4585881; doi:10.1038/srep14454)
Supplement: Supplementary Information [file srep14454-s1.pdf]

Supplementary Information

Latitudinal variation of leaf stomatal traits from species to community level in forests: linkage with ecosystem productivity

Ruili Wang[1,2], Guirui Yu[1,*], Nianpeng He[1,*], Qiufeng Wang[1], Ning Zhao[1,2], Zhiwei Xu[1,2] & Jianping Ge[3]

[1] Synthesis Research Center of Chinese Ecosystem Research Network, Key Laboratory of Ecosystem Network Observation and Modeling, Institute of Geographic Sciences and Natural Resources Research, Chinese Academy of Sciences, Beijing 100101, China

[2] University of Chinese Academy of Sciences, Beijing 100049, China

[3] College of Life Sciences, Beijing Normal University, Beijing 100875, China

*Correspondence and requests for materials should be addressed to G.Y. (yugr@igsnrr.ac.cn) and N.H. (henp@igsnrr.ac.cn).

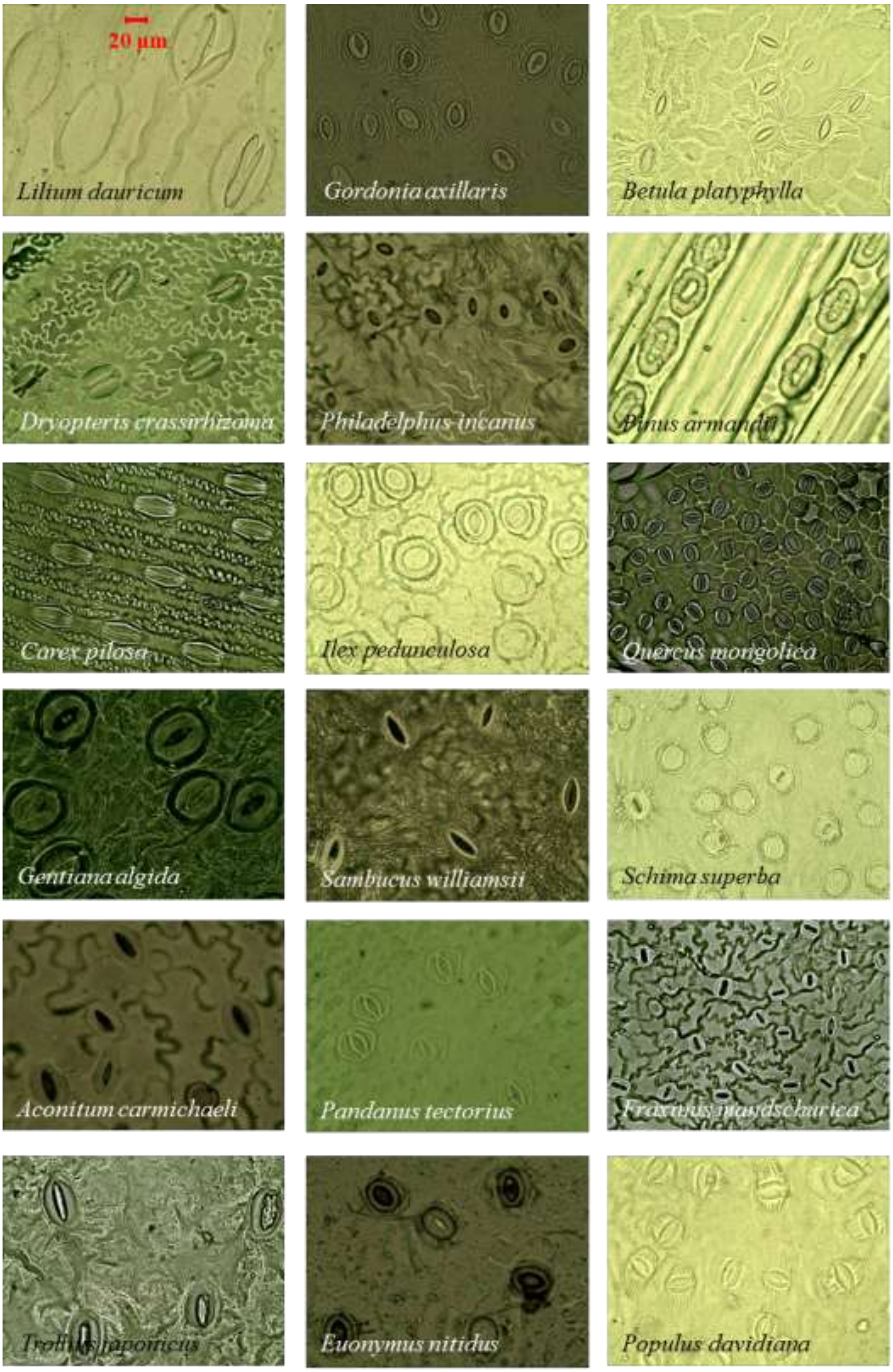

Supplementary Figure S1. Micrographs of the abaxial leaf surface of herbs (left panel), shrubs (middle panel) and trees (right panel). All images are taken at ×400 magnification. Sale bar = 20 μm.

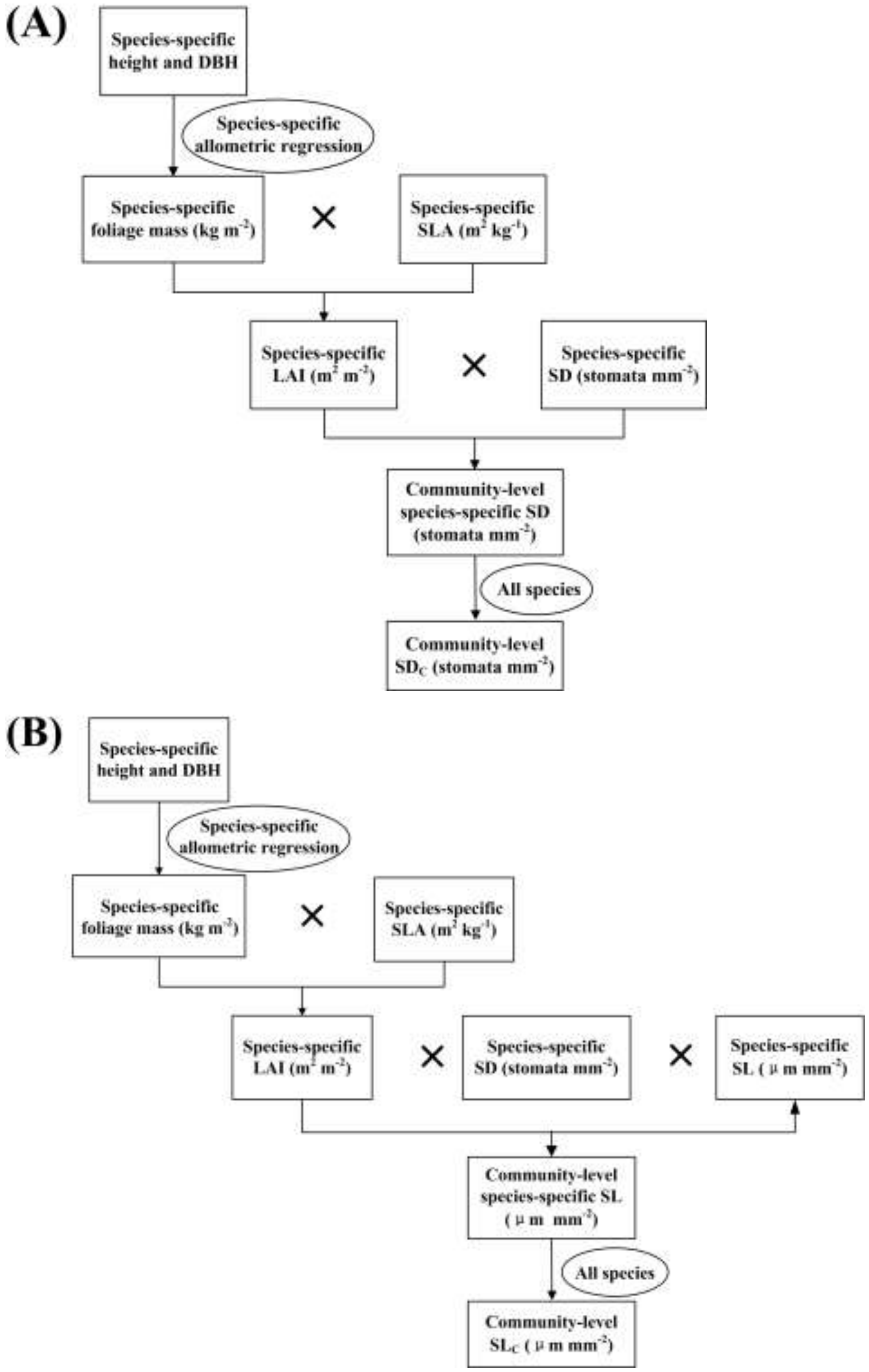

Supplementary Figure S2. Frameworks to calculate the community-level stomatal density (SD_C, A) and stomatal length (SL_C, B), respectively.

DBH, diameter at breast height for trees, and basal stem diameter for shrubs; SLA, specific leaf area; LAI, leaf area index.

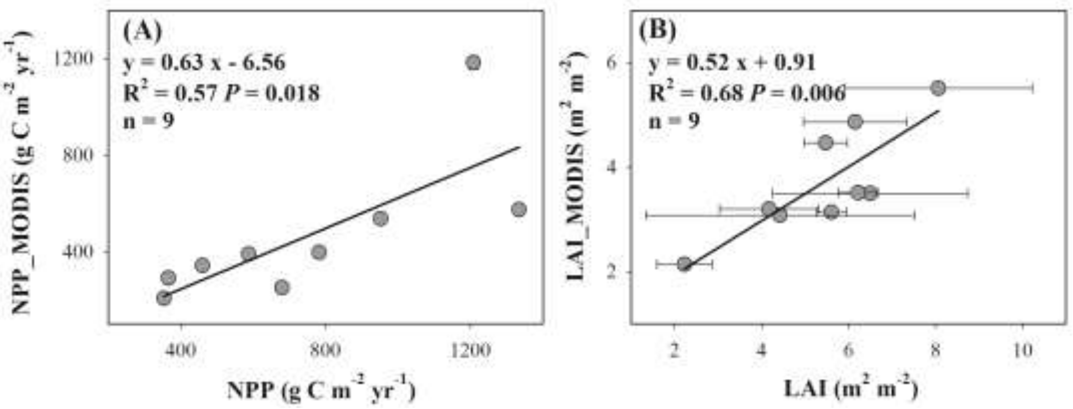

Supplementary Figure S3. Relationships between net primary productivity (NPP, A) and leaf area index (LAI, B) with the MODIS products in a 1 × 1 km grid. MODIS NPP data from 2000 to 2010 and LAI data from July to August of 2013 for nine sampling forests were selected and averaged for these time periods.

Supplementary Table S1. Description of the selected sampling sites.

Site	Latitude (°N)	Longitude (°E)	Altitude (m)	MAT (°C)	MAP (mm)	Soil N (mg g^{-1})	Soil C (mg g^{-1})	Soil type	Vegetation type
JF	18.74	108.86	809	23.15	2265.80	1.95	22.28	Laterite soil	Tropical monsoon rainforest
DH	23.17	112.54	240	21.83	1927.00	1.76	28.06	Lateritic red soil	Subtropical evergreen broadleaved forest
JL	24.58	114.44	562	18.22	1769.93	2.35	35.70	Red soil	Subtropical evergreen broadleaved forest
SN	31.32	110.50	1510	8.50	1446.71	3.76	41.89	Yellow brown soil	Mixed evergreen and deciduous broadleaved forest
TY	36.70	112.08	1668	5.98	644.38	2.56	45.10	Cinnamon soil	Temperate deciduous broadleaved forest
DL	39.96	115.42	972	6.55	539.07	3.12	38.93	Brown soil	Temperate deciduous broadleaved forest
CB	42.40	128.09	758	2.79	691.00	6.37	70.39	Dark brown soil	Temperate mixed forest
LS	47.19	128.90	401	0.01	648.34	4.59	77.02	Dark brown soil	Temperate mixed forest
HZ	51.78	123.02	850	-3.67	472.96	3.15	49.40	Grey forest soil	Cold-temperate coniferous forest

Note: JF, Jianfengling; DH, Dinghu; JL, Jiulian; SN, Shennongjia; TY, Taiyue; DL, Dongling; CB, Changbai; LS, Liangshui; HZ, Huzhong. MAT: mean annual temperature; MAP: mean annual precipitation; Soil N, soil total nitrogen concentration; Soil C, soil total carbon concentration.

Supplementary Table S2. Changes in stomatal density (SD) and stomatal length (SL) at the species and community levels for nine typical ecosystems.

Site	Species level			Community level		
	No. of species	SD_L (stomatal mm^{-2})	SL_L (μm)	No. of plots	SD_C (stomatal mm^{-2})	SL_C (μm mm^{-2})
JF	126	270.61 ± 13.26	22.65 ± 0.55	3	2685.39 ± 595.87	53986.19 ± 12086.04
DH	137	303.61 ± 16.83	25.35 ± 0.83	4	1718.80 ± 242.48	40592.08 ± 6506.17
JL	155	232.03 ± 11.03	26.73 ± 0.76	3	2050.8 ± 684.89	44830.91 ± 14837.56
SN	111	203.12 ± 14.85	30.91 ± 1.16	3	1761.58 ± 427.94	42255.25 ± 7850.03
TY	71	193.15 ± 14.72	29.40 ± 1.15	4	1533.89 ± 222.75	34461.42 ± 4287.30
DL	74	171.98 ± 13.11	29.46 ± 0.89	3	1632.50 ± 219.80	41637.87 ± 7507.44
CB	72	148.36 ± 13.69	33.98 ± 1.57	4	1752.85 ± 239.32	44934.78 ± 6301.82
LS	85	157.63 ± 12.26	31.95 ± 1.33	4	852.19 ± 267.14	26993.83 ± 8240.57
HZ	73	187.27 ± 12.46	28.80 ± 1.36	4	352.19 ± 120.75	10132.19 ± 3439.08
All	760	219.15 ± 5.56	28.16 ± 0.40	32	1497.09 ± 149.73	36032.13 ± 3204.86

Note: Data represent means ± 1 standard error. The full names of the sampling sites are given in Supplementary Table S1.

Supplementary Table S3. Standardized major axis (SMA) regression analyses for log-log transformed relationships between stomatal density (SD_L) and stomatal length (SL_L) of different groups.

Group	n	R^2	*P*	Slope	Slope CI	Intercept	Heterogeneity of slope	Shift in elevation	Common slope
Growth type									
Tree	294	0.37	< 0.001	-0.53[a]	(-0.58, -0.49)	2.65			
Shrub	273	0.39	< 0.001	-0.47[a]	(-0.52,-0.43)	2.46	*P* = 0.001		
Herb	310	0.46	< 0.001	-0.62[b]	(-0.67, -0.57)	2.78			
Leaf habit									
Evergreen	158	0.25	< 0.001	-0.48	(-0.55, -0.42)	2.51[a]	*P* = 0.872	*P* = 0.004	-0.49
Deciduous	121	0.35	< 0.001	-0.49	(-0.57, -0.42)	2.56[b]			

Note: Number of observations (n), coefficients of determination (R^2) and significance values (*P*) of each bivariate relationship are shown. 95% confidence intervals (CI) of SMA slopes are shown in parentheses. In growth type case, SMA tests for common slopes revealed significant differences among different groups ($P < 0.05$), then post-hoc multiple comparisons of slopes among growth groups were conducted. Superscript letters distinguish plant functional groups that differ significantly ($P < 0.05$). In leaf habit case, SMA tests for common slopes revealed no significant differences between the two plant groups ($P > 0.05$). Hence, the common slope was provided, and difference in elevation (i.e. y-axis intercept) between SMAs were then tested.

Supplementary Table S4. Pearson's correlations of selected climatic and soil variables. MAT, mean annual temperature; MAP, mean annual precipitation; Soil N, soil total nitrogen concentration; Soil P, soil total phosphorus concentration; SWC, soil water content. **, P < 0.01.

	Latitude	MAT	MAP	Insolation	Soil N	Soil P
MAT	-0.989**					
MAP	-0.936**	0.932**				
Insolation	-0.32	0.247	0.234			
Soil N	0.618	-0.631	-0.584	-0.227		
Soil P	0.564	-0.582	-0.569	-0.249	0.909**	
SWC	0.324	-0.336	-0.280	-0.167	0.863**	0.928**

Supplementary Table S5. Mixed-effect models for stomatal density (SD) and stomatal length (SL) at the species and community levels. Explanatory terms in bold are significant terms (*P* < 0.05). AIC, Akaike's Information Criterion value; PFT, plant functional type; MAT, mean annual temperature; MAP, mean annual precipitation; Soil N, soil nitrogen concentration; Soil P, phosphorus concentration; SWC, soil water content.

Dependent variable	Model	Explanatory terms	AIC
Log SD_L	Main-effect model	**PFT + MAT**	-17.27
		**PFT + MAP**	-12.82
		**PFT +** insolation	-1.36
		**PFT + Soil N**	-35.28
		**PFT + Soil P**	-22.99
		**PFT + SWC**	-18.18
		**PFT + MAT + Soil N**	-33.83
		**PFT + MAT + Soil P**	-23.40
		**PFT + MAT + SWC**	-23.64
		**PFT + MAP + Soil N**	-33.67
		**PFT + MAP + Soil P**	-22.49
		**PFT + MAP + SWC**	-22.84
	Interaction-effect model	**PFT + MAT + interaction**	-18.88
		**PFT + MAP + interaction**	-19.11
		**PFT + Soil N + interaction**	-40.05
		**PFT + Soil P + interaction**	-30.45
		**PFT + SWC + interaction**	-22.15
		**PFT + MAT + Soil N + 3 interactions**	-45.29
		**PFT + MAT + Soil P +** 3 interactions	-27.58
		**PFT + MAT + SWC +** 3 interactions	-23.32
		**PFT + MAP + Soil N + 3 interactions**	-43.90
		**PFT + MAP + Soil P +** 3 interactions	-30.36
		**PFT + MAP + SWC + 3 interactions**	-29.59
	Final model	**PFT + MAT + Soil N + 3 interactions**	-45.29
Log SL_L	Main-effect model	**PFT + MAT**	-1094.54
		**PFT +** MAP	-1085.54
		**PFT +** insolation	-1085.49
		**PFT + Soil N**	-1087.10
		**PFT + Soil P**	-1091.49
		**PFT + SWC**	-1090.00
		**PFT + MAT + Soil N**	-1092.57

		**PFT + MAT + Soil P**	-1089.70
		**PFT + MAT + SWC**	-1089.57
	Interaction-effect model	**PFT + MAT +** interaction	-1080.79
		**PFT + Soil N +** interaction	-1089.39
		**PFT + Soil P +** interaction	-1086.36
		**PFT + SWC +** interaction	-1089.45
		**PFT + MAT + Soil N +** 3 interactions	-1084.91
		**PFT + MAT + Soil P +** 3 interactions	-1085.24
		**PFT + MAT + SWC +** 3 interactions	-1087.41
	Final model	**PFT + MAT**	-1094.54
Log SD_C	Main-effect model	**MAT**	-22.84
		**MAP**	-13.66
		**insolation**	-10.12
		Soil N	-6.17
		Soil P	-6.33
		SWC	-5.81
		**MAT +** insolation	-22.14
		**MAP +** insolation	-14.08
	Final model	**MAT**	-22.84
Log SL_C	Main-effect model	**MAT**	-69.25
		**MAP**	-60.59
		insolation	-55.02
		Soil N	-53.13
		Soil P	-53.21
		SWC	-52.37
	Final model	**MAT**	-69.25

Supplementary Table S6. Nested random-effect analysis of variance (ANOVA) results for the partitioning the variance of species-level stomatal density (SD_L) and length (SL_L) into the within-site, among-site and residual components.

	Within-site	Among-site	Residuals	No. of observations	No. of sites
Log SD_L	54.1%	13.0%	32.9%	904	9
Log SL_L	53.3%	10.3%	36.4%	870	9

Supplementary Table S7. Summary of general linear models (GLM) of ecosystem net primary productivity (NPP).

Explanatory variable	*df*	MS	SS%	*F*	*P*
SD_C	1	206.74	51.04	6.35	0.045
SL_C	1	3.11	0.77	0.10	0.77
Residual	6	32.54	48.20		

Note: The explanatory variables include community-level stomatal density (SD_C) and stomatal length (SL_C). *df*, degrees of freedom; MS, mean squares; SS%, percentage of sum of squares explained.

Supplementary Table S8. Allometric biomass equations used in this study. These allometric equations were obtained from Chinese Ecosystem Research Net (CERN) database (http://159.226.111.42/pingtai/cernc/index.jsp), published studies, and our previous field measurements (unpublished data).

Development of allometric biomass equations

To develop these allometric equations, destructive tree sampling was carried out in each site. The dominant, co-dominant, intermediate trees, and understory shrubs were destructively sampled during July and August when the biomass reached its maximum. Stems were cut at the soil surface. Total tree height (from ground to base of the terminal bud, H, m), length of live crown, diameter at breast height (basal stem diameter for shrub, D, cm) were measured and recorded immediately. Stems were cut into 1 m sections and weighed. All live branches from each canopy position were divided into foliage, branches, and reproductive tissues. The entire root system of sample trees was excavated using a pulley device and manually digging approach. The fresh mass of each component was dried to a constant mass at 70 ℃ and weighted by electronic balance. Allometric equations between component biomass and independent variables (D, H) were developed by following these below relationships:

$$W = a(D^2H)^b \quad (1)$$

$$W = aD^b \quad (2)$$

where W is component biomass (kg).
